# SARS-CoV-2 Entry Related Viral and Host Genetic Variations: Implications on COVID-19 Severity, Immune Escape, and Infectivity

**DOI:** 10.3390/ijms22063060

**Published:** 2021-03-17

**Authors:** Szu-Wei Huang, Sheng-Fan Wang

**Affiliations:** 1 Model Development Section, Basic Research Laboratory, Center for Cancer Research, National Cancer Institute, Frederick, MD 21702, USA; szu-wei.huang@nih.gov; 2 Center for Tropical Medicine and Infectious Disease, Kaohsiung Medical University, Kaohsiung 80708, Taiwan; 3 Drug Development and Value Creation Research Center, Kaohsiung Medical University, Kaohsiung 80708, Taiwan; 4 Department of Medical Laboratory Science and Biotechnology, Kaohsiung Medical University, Kaohsiung 80708, Taiwan; 5 Clinical Microbiology Laboratory, Department of Laboratory Medicine, Kaohsiung Medical University Hospital, Kaohsiung Medical University, Kaohsiung 80708, Taiwan; 6 Department of Medical Research, Kaohsiung Medical University Hospital, Kaohsiung Medical University, Kaohsiung 80708, Taiwan

**Keywords:** SARS-CoV-2, COVID-19, spike glycoprotein, mutation, genetic variation, ACE2, variants, immune escape, transmissibility

## Abstract

Severe acute respiratory syndrome coronavirus 2 (SARS-CoV-2) has evolved to display particular patterns of genetic diversity in the genome across geographical regions. These variations in the virus and genetic variation in human populations can determine virus transmissibility and coronavirus disease 2019 (COVID-19) severity. Genetic variations and immune differences in human populations could be the driving forces in viral evolution. Recently emerged SARS-CoV-2 variants show several mutations at the receptor binding domain in the spike (S) glycoprotein and contribute to immune escape and enhanced binding with angiotensin 1-converting enzyme 2 (ACE2). Since ACE2 and transmembrane protease serine 2 (TMPRSS2) play important roles in SARS-CoV-2 entry into the cell, genetic variation in these host entry-related proteins may be a driving force for positive selection in the SARS-CoV-2 S glycoprotein. Dendritic or liver/lymph cell-specific intercellular adhesion molecule (ICAM)-3-grabbing non-integrin is also known to play vital roles in several pathogens. Genetic variations of these host proteins may affect the susceptibility to SARS-CoV-2. This review summarizes the latest research to describe the impacts of genetic variation in the viral S glycoprotein and critical host proteins and aims to provide better insights for understanding transmission and pathogenesis and more broadly for developing vaccine/antiviral drugs and precision medicine strategies, especially for high risk populations with genetic risk variants.

## 1. Introduction

The recently identified severe acute respiratory syndrome coronavirus 2 (SARS-CoV-2) is responsible for the pandemic of coronavirus disease 2019 (COVID-19), which began at the end of 2019 and is ongoing [1,2]. The number of confirmed cases continues to rise rapidly worldwide, with nearly 78% of confirmed cases in the Americas (44.5%) and Europe (34%) (calculated until 22 February 2021) (World Health Organization (WHO) COVID-19 report, https://covid19.who.int/, accessed on 22 February 2021). According to a meta-analysis, the fatality rate of COVID-19 is around 3% [3].

SARS-CoV-2 is the third coronavirus to cause a pandemic, the other two being SARS-CoV [4] and Middle East Respiratory Syndrome Coronavirus (MERS-CoV) [5] in 2003 and 2012, respectively. Genetic evidence from the SARS-CoV-2 genome shows high identity with two bat-derived SARS-like coronaviruses, bat-SL-CoVZC45 (87.6%) and bat-SL-CoVZXC21 (87.5%) [6]. Due to the lack of antiviral drug and vaccine selection pressure, the current genetic diversity patterns of SARS-CoV-2 in different geographical regions may be associated with genetic variation in populations, with increasing genetic diversity in the virus attributed to natural selection driven by long periods of an evolutionary arms race between host and virus [7]. Several studies have demonstrated that there is a positive correlation between host cell genetic variation and the susceptibility to different viruses [8,9,10]. Older age, male sex, and some co-morbidities have been found to be risk factors associated with COVID-19 severity, however, those risk factors do not fully explain the differences between asymptomatic, mild, and severe patients [11]. A recent genome-wide association study by Ellinghaus and colleagues showed that rs1138592 and rs657152 genetic variants were significantly associated with respiratory failure in severe COVID-19 patients. Notably, rs65712 is located at the *ABO* blood group gene, and Ellinghaus and colleagues further confirmed that patients with blood group A showed higher risk than others [12]. Another study by Zeberg and Pääbo found that the core haplotype in chromosome 3 is strongly associated with COVID-19 severity. The frequency of this haplotype was found to vary between South Asian (30%), European (8%), admixed American (4%), and East Asian (almost absent) populations [13]. However, future study is required to investigate the significance of this variation on COVID-19 severity. It is believed that human genetic variation can result in different responses to SARS-CoV-2 infection, even with the same age, sex, and health status. SARS-CoV-2 has evolved to contain cumulative mutations in its genome, with the most highly mutated regions being ORF1ab, spike, and nucleocapsid genes. It is inferred that positive selection contributes to the evolution of SARS-CoV-2 [14,15]. Several recently emerged SARS-CoV-2 variants, B.1.1.7 lineage (a.k.a. 20B/501Y.V1 Variant and VUI202012/01), B.1.351 lineage (a.k.a. 20C/501Y.V2), P.1/P.2 lineages (descendent of B.1.1.28), and B.1.429, have been found responsible for the dramatic increase of infections in the United Kingdom [16], South Africa [17], Brazil [18], and North America [19], respectively. Viral genome analysis showed these variants to carry multiple mutations in the S glycoprotein, including some at the receptor binding domain (RBD). Some of these mutations are believed to be the result of adaptive evolution and have biological importance. The direct impacts of the mutations in the S glycoprotein of SARS-CoV-2 include affecting the viral transmissibility through interaction with the host cell binding receptor and contributing to the immune escape through changes in the RBD. The most important host proteins involved in SARS-CoV-2 entry have been identified as angiotensin 1-converting enzyme 2 (ACE2) and cell-surface associated transmembrane protease serine 2 (TMPRSS2). Genetic variations in *ACE2* and *TMPRSS2* may provide the driving force for viral evolution, therefore causing positive selection for these emerging mutations in the SARS-CoV-2 S glycoprotein. Additionally, dendritic or liver/lymph cell-specific intercellular adhesion molecule (ICAM)-3-grabbing non-integrin (DC/L-SIGN) has been known to play vital roles for several pathogens, including SARS-CoV [20]. Therefore, the genetic variation of these host proteins may also affect susceptibility to SARS-CoV-2. Investigating the correlations between genetic variation in populations and viral infectivity or clinical outcomes could provide great insights for developing precision medicine strategies.

In this review, we aim to compile knowledge and current advances on the impacts of genetic variations in the viral S glycoprotein and critical host proteins on the susceptibility to SARS-CoV-2 infection and immune escape. This understanding is crucial for controlling the pandemic through enhanced surveillance and vaccine development.

## 2. Brief Introduction to Coronavirus Proteins and Mutations

**Figure 1:**
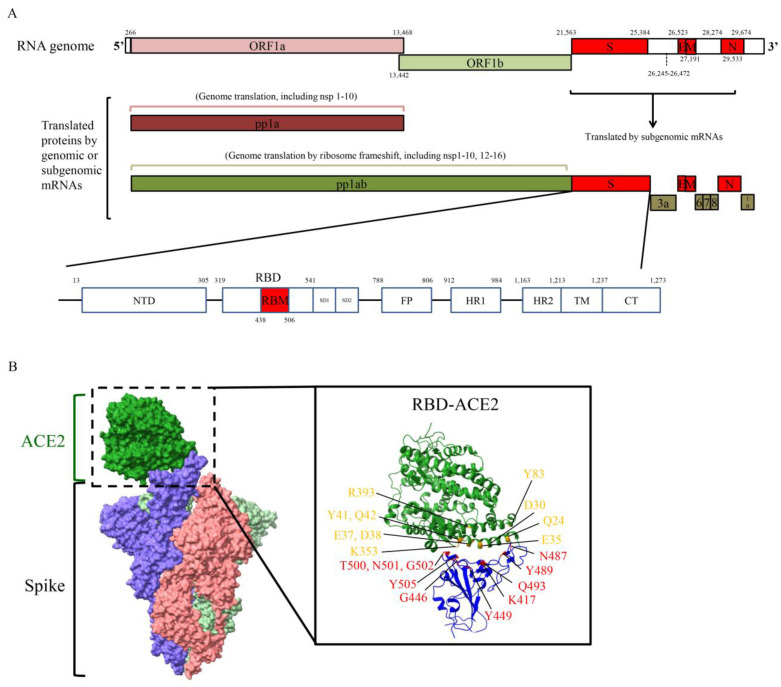
Severe acute respiratory syndrome coronavirus 2 (SARS-CoV-2) genomic organization and structure of S glycoprotein. (**A**) The ORF1a and ORF1b genes can be translated to polyprotein 1a (pp1a) and polyprotein 1ab (pp1ab, -1 ribosomal frameshift). Pp1a and pp1ab can be processed into several functional non-structural proteins (nsps). Structural genes encode four structure proteins, S glycoprotein, envelope protein (E), membrane protein (M), and nucleocapsid protein (N). Several accessory proteins are encoded in the end of genome, include ORF3a, ORF6, ORF7, ORF8, and ORF10. NTD, *N*-terminal domain; SD1 and SD2, subdomain 1 and 2; FP, fusion peptide; HR1 and HR2, heptad repeat 1 and 2; TM, transmembrane region. (**B**) Structure of SARS-CoV-2 S glycoprotein bound to angiotensin 1-converting enzyme 2 (ACE2). S glycoprotein consists of three S glycoprotein monomers which are shown in blue, pink, and light green. ACE2 is shown in green. The interface of SARS-CoV-2 receptor binding domain (RBD)-receptor binding motif (RBM) and ACE2 is enlarged in right panel. The amino acid positions of RBD and ACE2 responsible for binding are shown in yellow (ACE2) and red (RBD). Structure depicting S glycoprotein bound to ACE2 using PDB: 7A94. Structure depicting RBD bound to ACE2 using PDB: 6VW1. Figures are generated by UCSF ChimeraX software.

SARS-CoV-2 is one of the coronaviruses (CoV) and is an enveloped and positive-sense ssRNA (~30 kb) virus which belongs to the Betacoronavirus genus, Nidovirales order. Two replicase open reading frames (ORFs) encoded by ORF1a (~13.2 kb) and ORF1b (~8.1 kb) occupy at least two-thirds of the CoV genome (Figure 1A). The polyprotein ORF1ab (as known as pp1ab) is translated due to a −1 ribosomal frameshift upstream of the ORF1a stop codon [21]. Polyprotein ORF1a (as known as pp1a) and pp1ab can be further processed to 16 functional non-structural proteins (nsps) by self-produced nsp5 and nsp3 proteases. Nsp5 protease (also called 3C-like protease, 3CLpro, Mpro) contains a chymotrypsin-like fold and is responsible for processing nsp4 to nsp16, whereas nsp3 papain-like protease (PLpro) is responsible for processing nsp1 to nsp4 [22]. SARS-CoV nsps have been well studied and characterized for their involvement in the different steps of the virus replication cycle [22].

Four structural protein genes are located in the *C*-terminal region of the CoV genome. The SARS-CoV-2 S glycoprotein contains a furin recognition cleavage site (polybasic cleavage site, PRRAR) which provides efficient proteolytic processing into S1 and S2 [23]. Additionally, recent evidence has shown that the furin cleavage site of the SARS-CoV-2 S glycoprotein plays a critical role in regulating viral replication and pathogenesis, which could be a potential therapeutic target against SARS-CoV-2 infection [24,25,26]. The SARS-CoV-2 RBD, located in the S1 domain, binds to the host cell ACE2 receptor, while S2 functions as the membrane fusion subunit [27,28]. Within the RBD, the receptor binding motif (RBM) is in close contact with ACE2. Several amino acid positions of the interface between ACE2 and RBM have been found to play important roles for binding through the formation of hydrogen bonds and salt bridges (Figure 1B). The envelope (E) protein is involved in virion production and pathogenesis [29], and the membrane (M) protein plays a pivotal role in mediating virus assembly and budding. In addition, the M protein interacts with the viral nucleocapsid (N) protein for viral RNA packaging and recruits other structural proteins to the endoplasmic reticulum (ER)-Golgi-intermediate compartment [30,31]. The N protein encapsulates and protects the CoV genome in the virion and then enters the host cells to promote viral replication [32,33]. Several accessory proteins can be found in CoV, however, though they can affect viral viability and pathogenesis, evidence has shown that accessory proteins are not essential for viral replication [34].

## 3. Impacts of Mutations on the SARS-CoV-2 S Glycoprotein

The first step of CoV infection of a target cell is the binding of the viral RBD of the S glycoprotein to the cell membrane receptor, ACE2 (Figure 1B). The SARS-CoV-2 RBD and ACE2 binding structure is nearly identical to that of the SARS-CoV RBD [35,36]. There are several studies which have shown that SARS-CoV-2 is more infectious than SARS-CoV, which may explain why SARS-CoV-2 has caused a more severe pandemic than SARS-CoV [37,38,39,40]. Three mechanisms which have been proposed to potentially play a role in this increased SARS-CoV-2 infectivity are: (1) higher binding affinity of RBD to ACE2, (2) less exposed RBD (immune escape), and (3) pre-activation by furin (enhanced viral entry) [41]. Other than ACE2, another protein which has been shown to be important in SARS-CoV-2 entry into the cell is TMPRSS2. The cleavage ability of TMPRSS2 to prime the S glycoprotein during viral attachment plays a vital role in SARS-CoV-2 entry into the cell [27]. Evidence has shown that the furin processing region (amino acid position 675 to 692) has the highest mutation density (number of distinct mutations in the region) [42]. Mutations which occur in this region may provide an advantage to the virus, allowing it to utilize a large number of host proteases to enhance infectivity. The S glycoprotein has also been shown to play pivotal roles in identifying host specificity, viral pathogenesis, and inducing human neutralizing antibodies. Viral surface proteins have been shown as promising targets to generate therapeutic or prevention purpose antibodies [43]. Current studies have focused on the mutation at the RBD (residues 319 to 541) in the SARS-CoV-2 S glycoprotein. Within the interface of the S glycoprotein (RBM) and ACE2, several mutations have been identified.

The continuous transmission of SARS-CoV-2 has caused rapid accumulation of mutations in the S glycoprotein across geographical regions. One of the predominant mutations, D614G, was found to be circulating and rapidly spreading outside China in the early pandemic. The recently emerged variants in the United Kingdom (B.1.1.7), South Africa (B.1.351), North America (B.1.429), and Brazil (B.1.1.28) exhibit several mutations in the S glycoprotein, especially in the RBD. B.1.1.7 carries eight mutations in the S glycoprotein which include H69/V70 deletion (ΔH69/V70), Y144 deletion (ΔY144), N501Y, A570D, P681H, T716A, S982A, and D1118H. B.1.351, recently found in South Africa, carries three mutations located in the RBD region which are K417N, E484K, and N501Y. B.1.1.28 (P.1 and P.2 lineages) in Brazil exhibits a different pattern of mutations. The P.1 lineage has the same RBD mutations as South Africa, whereas the P.2 lineage has independently accrued the spike E484K mutation [44]. B.1.429, which has recently been found to be spreading rapidly in California, USA, includes three mutations in the S glycoprotein, S13I and W152C in the S1 domain and L452R in the RBD [19]. Those variants have caused a severe increase in SARS-CoV-2 infections since December 2020. Notably, the N501Y mutation, which is located in the RBD and has been found in most of the variants, is believed to enhance the transmissibility of SARS-CoV-2. The aforementioned variants all have the D614G mutation, though this is expected due to the predominance of D614G since the early pandemic. Other than the mutations found in those variants, several mutations in S glycoprotein have been found sporadically and contribute to the immune escape and the transmissibility of SARS-CoV-2.

### 3.1. Impacts of Mutations in the S Glycoprotein on Transmissibility and Infectivity

The impact of D614G on viral transmissibility has been widely studied due to its emergence in the early pandemic and its worldwide presence. The D614G mutation is of rising concern, as it has the potential to affect SARS-CoV-2 infectivity through changes to RBD structure, S1/S2 subunit interaction, viral entry, and immune response [45]. Becerra-Flores and colleagues have found that patients infected with SARS-CoV-2 containing the D614G mutation have a higher case fatality rate [46]. The effects of the D614G mutation on the SARS-CoV-2 S glycoprotein have been comprehensively investigated by several studies. First, this mutation has been found to be associated with higher viral load in the upper respiratory tract in patients, further confirmed in pseudotyped experiments and animal models [45,47,48]. Second, a detailed structure analysis showed that the D614G mutation shifts the conformation of the S glycoprotein to be more open, therefore contributing to enhanced ACE2 binding and fusion efficiency [49]. This conformation change of the S glycoprotein has been found to be important for SARS-CoV-2 binding with ACE2 [50]. Third, the D614G mutation can decrease S1 shedding, which indicates enhanced efficiency of processing by furin-like proprotein convertase [51]. However, the D614G mutation did not show resistance to neutralizing antibodies [52]. A recent report indicated that the D614G mutation can potentially affect the glycosylation at residue 616 which may be able to enhance virulence through DC/L-SIGN binding in dendritic cells [53]. Notably, D614G combined with other mutations in S glycoprotein exhibits more infectivity in different cell lines [54]. As there was no available treatment or vaccination selective pressure in SARS-CoV-2 infection in the early pandemic, how the D614G mutation occurred and became predominant outside China is not clear. Our previous study showed that the D614G mutation is significantly associated with the differences in ACE2 expression levels in populations [55]. This study indicates that populations with lower ACE2 expression, such as Europe and Africa, provide the environment for selective pressure for SARS-CoV-2 adaptive evolution.

ΔH69/V70 are located at the *N*-terminal domain (NTD) of the S glycoprotein. The ΔH69/V70 deletion has been found globally, however, tracking of SARS-CoV-2 sequences has shown it to be mainly circulating in Europe. A single round infectivity experiment showed that SARS-CoV-2 carrying either ΔH69/V70 or ΔH69/V70 combined with N501Y can enhance infectivity in 293T/hACE2 cells. Additionally, a virus carrying the ΔH69/V70 mutation exhibits more S glycoprotein incorporated in the virion [56]. A recent study showed that ΔH69/V70 with D796H in the S glycoprotein can potentially contribute to immune escape in immunocompromised patients, while D796H itself decreased the infectivity but contributed to the reduction of susceptibility to neutralizing antibodies [57]. The loss of infectivity caused by D796H could be compensated for in cases where it co-occurs with ΔH69/V70. ΔH69/V70 also frequently co-occurs with N439K or Y453F, which are located at the RBD of the SARS-CoV-2 S glycoprotein. The binding affinity of Y453F with ACE2 is controversial, however, it is seen to contribute to the immune escape for neutralizing antibodies and human convalescent sera [58,59,60]. The N439K mutation could enhance the binding affinity with ACE2 through the formation of a new salt bridge and has resistance to some neutralizing antibodies and human convalescent sera [61].

S477N is located in the RBD and has been found to enhance binding with ACE2 [58]. The E484K mutation in the RBD of S glycoprotein is of rising concern due to its emergence in several current variants which cause severe transmission. A current study showed that E484K could enhance the binding with ACE2 through a conformational change of the S glycoprotein [62]. L452R, located at the RBD, has been shown to increase the infectivity by stabilizing the S glycoprotein and ACE2 interaction [63,64,65]. N501Y is another mutation of rising concern due to its co-occurrence in several current SARS-CoV-2 variants in the United Kingdom and South Africa. N501Y is also located in the RBD of the S glycoprotein and could potentially affect binding with ACE2. The N501Y mutation emerged in infected wild-type mice at early passage and is believed to be the result of adaptive evolution of the SARS-CoV-2 virus [66]. Studies using a comprehensive scanning approach [58] and in silico methods [67] have shown that the N501Y mutation can increase the binding affinity for ACE2. The resulting enhanced binding affinity may be due to additional hydrogen bonds with ACE2 at residues Y41 and K353 [68] and may contribute to a more open conformation of the RBD in the S glycoprotein [69]. The P681H mutation is juxtaposed to the furin processing site (amino acid position 682 to 685). The furin processing of the S glycoprotein into S1/S2 is an important step for virus fusion into cells [70], however, whether the P681H mutation could affect viral infectivity and efficiency of furin processing needs further investigation. V1176 is located at the stalk domain of the S glycoprotein. The flexible stalk domain is necessary for viral entry and fusion into the cells [71]. According to a molecular dynamics simulations analysis, the V1176F mutation could enhance the flexibility of the S glycoprotein by increasing motility and inducing compactness [72]. Additionally, evidence has shown that V1176F is associated with higher patient mortality [72,73]. The mutations N331Q and N343Q could disrupt the N-glycosylation site of the S glycoprotein and strongly decrease the viral infectivity, however, there is no current circulating SARS-CoV-2 carrying those mutations [54,74] (Table 1).

**Table 1:** Impacts of mutations in S glycoprotein in circulating SARS-CoV-2.

Mutation	S Region	Potential Effects	% of All Sequences (*n* = 498,134)	% of Mutation in Geographical Regions	Ref.
ΔH69/V70	S1 NTD	Usually co-occurs with other RBD mutations. Enhance the infectivity and compensate the decrease of infectivity due to emerge of RBD immune escape mutations, such as D796H.	17.3 (*n* = 86,033)	Asia (0.6) Europe (97.5) North America (1.6) South America (<0.1) Africa (0.1) Oceania (0.1)	[56,57]
ΔY144	S1 NTD	Loss of binding ability with neutralizing antibodies.	15.3 (*n* = 76,387)	Asia (0.6) Europe (97.8) North America (1.3) South America (<0.1) Africa (0.2) Oceania (0.1)	[75,76]
ΔL242/244	S1 NTD	Loss of binding ability with neutralizing antibodies.	0.2 (*n* = 1049)	Asia (2.3) Europe (25.2) North America (1.1) Africa (70.3) Oceania (1.1)	[75,76]
N331Q	RBD	Disrupt N-glycosylation site. Strong decrease infectivity.	NA	NA	[54,74]
N343Q	RBD	Disrupt N-glycosylation site. Strong decrease infectivity.	NA	NA	[54,74]
E406W	RBD	Immune escape the neutralization by REGN-COV2 cocktail (REGN10987+ REGN10933).	NA	NA	[77]
K417N/T/V	RBD	Immune escape the neutralization by several monoclonal antibodies (included LY-CoV016 and others). Decrease binding with ACE2.	0.3 (*n* = 1373)	Asia (1.9) Europe (29.9) North America (1.1) South America (7.6) Africa (58.3) Oceania (1.2)	[52,77]
N439K	RBM	Immune escape the neutralization by monoclonal antibodies (REGN10987 and others) and human convalescent sera. Enhance the binding with ACE2 through new forms a salt bridge with ACE2.	2.3 (*n* = 11,396)	Asia (1.1) Europe (98.1) North America (0.6) South America (<0.1) Africa (<0.1) Oceania (0.2)	[52,54,77]
N440D/K	RBM	Immune escape the neutralization by monoclonal antibodies (REGN10987 and others).	<0.1 (*n* = 3)	Europe (66.7) North America (33.3)	[52,77]
K444E/N	RBM	Immune escape the neutralization by monoclonal antibodies and human convalescent sera.	<0.1 (*n* = 32)	Asia (3.1) Europe (25) North America (68.8) Oceania (3.1)	[78]
G446D/V	RBM	Immune escape the neutralization by monoclonal antibodies and human convalescent sera.	<0.1 (*n* = 151)	Asia (2.6) Europe (72.2) North America (20.5) South America (1.3) Oceania (3.3)	[54,78]
N450K/Y/D	RBM	Immune escape the neutralization by monoclonal antibodies and human convalescent sera.	<0.1 (*n* = 47)	Asia (2.1) Europe (74.5) North America (21.3) Africa (2.1)	[78]
L452R	RBM	Immune escape the neutralization by monoclonal antibodies and human convalescent sera. Enhance the infectivity.	0.7 (*n* = 3722)	Asia (2.2) Europe (13) North America (82.4) South America (0.2) Africa (1.7) Oceania (0.5)	[54,65,78]
Y453F	RBM	Immune escape the neutralization by several monoclonal antibodies (REGN10933) and human convalescent sera.	0.2 (*n* = 1032)	Europe (97) North America (2.7) Africa (0.3)	[77]
A475V	RBM	Immune escape the neutralization by several monoclonal antibodies and human convalescent sera.	<0.1 (*n* = 118)	Asia (0.8) Europe (78) North America (16.1) South America (0.8) Africa (1.7) Oceania (2.5)	[52,54]
G476S	RBM	Immune escape the neutralization by monoclonal antibodies (H014).	<0.1 (*n* = 56)	Asia (7.1) Europe (55.4) North America (35.7) South America (1.8)	[54]
S477N	RBM	Broadly resistance to monoclonal antibodies, whereas sensitive to human convalescent sera. Enhance the binding with ACE2.	5.2 (*n* = 25,970)	Asia (0.2) Europe (60) North America (1.3) South America (0.3) Africa (0.7) Oceania (37.5)	[58,78]
T478I	RBM	Immune escape the neutralization by monoclonal antibodies and human convalescent sera.	<0.1 (*n* = 142)	Europe (93) North America (5.6) Africa (1.4)	[54,78]
P479S	RBM	Immune escape the neutralization by monoclonal antibodies.	<0.1 (*n* = 175)	Asia (2.3) Europe (87.4) North America (9.7) South America (0.6)	[78]
E484K	RBM	Broadly Immune escape the neutralization by antibodies and human convalescent sera. Enhance the binding with ACE2.	0.5 (*n* = 2483)	Asia (2.2) Europe (30.8) North America (17.8) South America (14.5) Africa (33.8) Oceania (0.8)	[52,62,76,78]
F486L	RBM	Immune escape the neutralization by monoclonal antibodies (REGN10933, 2B04, and 1B07).	<0.1 (*n* = 17)	Europe (82.4) North America (17.6)	[77,78]
Y489H	RBM	Immune escape the neutralization by monoclonal antibodies (REGN10933).	<0.1 (*n* = 4)	Europe (50) North America (50)	[77]
Q493K	RBM	Immune escape the neutralization by monoclonal antibodies (REGN10933).	<0.1 (*n* = 6)	Europe (33.3) North America (66.7)	[77]
P499L	RBM	Immune escape the neutralization by monoclonal antibodies and human convalescent sera.	<0.1 (*n* = 3)	Europe (33.3) North America (33.3) South America (33.3)	[78]
N501Y	RBM	Enhance binding with ACE2 through new forms of hydrogen bond and open conformation of RBD.	16 (*n* = 79,510)	Asia (0.6) Europe (96.8) North America (1.1) South America (0.2) Africa (1.2) Oceania (0.2)	[52,56,58,67]
D614G	S1 CTD	Enhance infectivity through changes to RBD structure, S1/S2 subunit interaction, viral entry, and immune response.	Predominant circulating worldwide	Predominant circulating worldwide	[45,47,48,49,50,51,52,54]
P681H	S2	Potential affect the furin processes and fusion.	16.5 (*n* = 81,974)	Asia (0.8) Europe (94) North America (4.8) South America (<0.1) Africa (0.2) Oceania (0.2)	[79]
D796H	S2	Co-occurring with ΔH69/V70 in immunocompromised patients with chronic infection treatment. Decrease infectivity. Reduction in susceptibility to non-RBD specific antibodies.	<0.1 (*n* = 115)	Asia (1.7) Europe (81.7) Africa (5.2)	[57]
V1176F	S2	Associates with COVID-19 patient mortality and may enhance the flexibility of stalk domain of S glycoprotein.	0.4 (*n* = 1,886)	Asia (1.7) Europe (18.1) North America (17.3) South America (61.3) Africa (0.6) Oceania (0.8)	[72,73]

A total of 498,134 SARS-CoV-2 sequences from the Global Initiative on Sharing Avian Influenza Data (GISAID) database (https://www.gisaid.org/, accessed on 16 February 2021), from 1 January 2020 to 16 February 2021, were analyzed to determine the percentage of mutations in different geographical regions. RBD, receptor binding domain; RBM, receptor binding motif; NTD: *N*-terminal domain; NA: not available.

### 3.2. Impacts of Mutations in the S Glycoprotein on Immune Escape Ability

The ΔY144 and the L242/L244 deletions (ΔL242/244) are located at the NTD of the S glycoprotein and show a loss of binding ability with neutralizing antibodies [75,76]. Starr and colleagues mapped the mutations in the RBD of SARS-CoV-2 which could escape neutralization by the antibodies used to treat COVID-19 patients, Regeneron’s REGN-COV2 cocktail (consisting of two antibodies, REGN10933 and REGN10987, emergency use authorization for treatment of COVID-19) and Eli Lilly’s LY-CoV016 antibody (also known as CB6 or JS016, phase 3 clinical trials). They found that E406W can escape the neutralization by the REGN-COV2 cocktail. K417N can escape the neutralization by several monoclonal antibodies including LY-CoV016. N439K and N440D can escape the neutralization by the REGN10987 antibody. Y453F, F486L, Y489H, and Q493K also escape the neutralization by REGN10933 [77]. K417N is one of the major mutations found in B.1.351 which has also been recently shown to escape the neutralization by monoclonal antibodies [52,77,80]. A recent study used free energy perturbation calculations to show that the combination of N501Y and K417N could enhance the binding with ACE2 while dramatically decreasing the binding with antibodies [81]. The E484K mutation, located in the RBD, not only enhanced binding with ACE2, it also exhibited strong or moderate resistance to several human neutralizing antibodies and human convalescent sera [52,76,78,80,82,83], which indicates this mutation is important in the viral evolution to escape neutralizing antibodies. L452R can also reduce the sensitivity to several antibodies and human convalescent sera [54,78]. Several rare mutations (<0.1%) have been found to contribute to the immune escape of neutralization by monoclonal antibodies and human convalescent sera, including N440D, K444N, G446D/V, N450K/Y/D, A475V, G476S, T478I, P479S, F486L, Y489H, Q493K, P499L, and D796H [54,57,77,78] (Table 1).

### 3.3. Geographical Distribution of Mutations in S Glycoprotein

**Figure 2:**
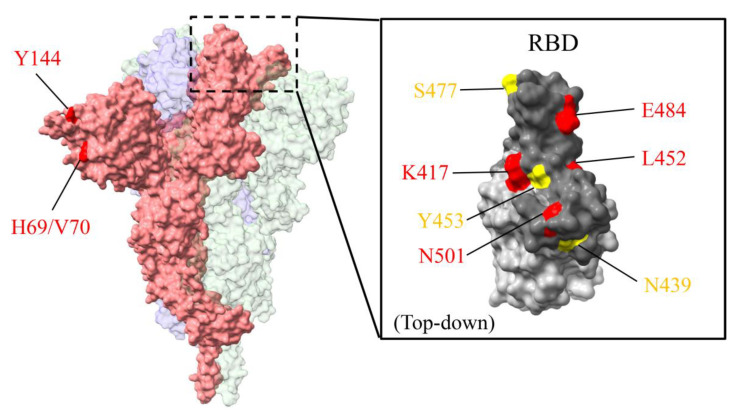
Structure of severe acute respiratory syndrome coronavirus 2 (SARS-CoV-2) S glycoprotein with current emerged mutations. Mutations existing in more than 1% of total sequences are shown. One monomer of S glycoprotein is shown in pink. The structure of the receptor binding domain (RBD) is enlarged in right panel and receptor binding motif (RBM) is shown in dark gray. Mutations in currently emerged SARS-CoV-2 variants are labeled in red, whereas others are in yellow. Structure depicting S glycoprotein using PDB: 6XR8. Structure depicting RBD using PDB: 7BWJ. Figures are generated by UCSF ChimeraX software.

There are several mutations which appear in more than 0.1% of sequences circulating across geographical regions, including ΔH69/V70 and Δ144 in S1 NTD; K417N/T/V, N439K, L452R, Y453F, S477N, E484K, and N501Y in RBD; D614G in S1 CTD; P681H and V1176F in S2. ΔH69/V70, Δ144, N439K, Y453F, and N501Y are found circulating mainly in Europe. K417N/T/V is carried by B.1.351 and B.1.1.28 which are mainly circulating in Africa and Europe. L452R is carried by B.1.429 which is mainly circulating in North America and Europe. S477N is mainly circulating in Europe and Oceania. E484K is carried by several current circulating variants and is found in Africa (33.8%) and Europe (30.8%), however, there are also more than 10% distributed in North and South Americas (Figure 2). P681H is carried by B.1.1.7 and is mainly found circulating in Europe. Notably, SARS-CoV-2 sequences carrying only P681H (excluded B.1.1.7) make up 4.8% of those circulating in North America. V1176F is mainly circulating in South America, however, there are more than 10% of SARS-CoV-2 sequences carrying this mutation in Europe and North America (Table 1).

Several functionally important mutations have been found to be circulating across geographical regions and co-occurring with other variants. E484K is one of the most concerning mutations which exhibits increased dynamics in several severe transmission regions. Recent evidence has shown that SARS-CoV-2 carrying E484K or K417N (less prevalent than E484K) in the S glycoprotein could contribute to broad immune escape from monoclonal antibodies and human convalescent sera [52,76]. The current SARS-CoV-2 variants exhibit high transmissibility and immune escape ability as a result of several co-occurring mutations in the S glycoprotein. Increased transmissibility is conferred by ΔH69/V70 and N501Y in B.1.1.7, E484K and N501Y in B.1.351, E484K in B.1.1.28, and L452R in B.1.429. Immune escape is contributed to by ΔY144 in B.1.1.7, E484K and K417N in B.1.351, E484K in B.1.1.28, and L452R in B.1.429.

## 4. Impacts of Genetic Variability of ACE2, TMPRSS2, and DC/L-SIGNs in SARS-CoV-2 Infectivity and Pathogenicity

### 4.1. ACE2 Genetic Variation and SARS-CoV-2 Infection

The *ACE2* gene contains 18 exons located in chromosome X. ACE2 consists of three domains: (1) *N*-terminal peptidase domain (residues 19-615), (2) C-terminal collectrin-like domain (CLD, residues 616-768), and (3) end with a hydrophobic transmembrane region and an intracellular segment of 43 residues [84,85]. ACE2 belongs to the family of angiotensin converting enzymes (ACE) members. ACE is a widely distributed protein which converts angiotensin (Ang) I (inactive form) to AngII (activate form). This conversion is known to play a vital role in several biological functions, such as controlling blood pressure [86,87], regulating water and sodium absorption in the kidneys [88], and mediating cell proliferation [89]. ACE2 has been demonstrated to be involved in regulating heart function, hypertension (HT), diabetic heart disease, and dyslipidemian [90]. Several studies have shown that polymorphisms of *ACE2* are significantly associated with blood pressure in different populations [91,92]. Additionally, COVID-19 patients who have HT, heart disease, and diabetes are associated with severe infections and clinical outcomes [11,93].

A previous study on a group 2 coronavirus demonstrated that the correlation between viral receptor genetic variation and viral binding activity can affect host susceptibility [94]. Similarly, the relationship between human immunodeficiency virus type 1 (HIV-1) gp120 and the CD4 T cell co-receptor CCR5 is another well-known example of a receptor polymorphism affecting viral entry. Individuals carrying the CCR5Δ32 polymorphism (CCR5 contains 32 bp deletions) can block HIV-1 entry into host cells and prevent infection [9]. For CoV, the polymorphisms (three missenses and one deletion) of the functional receptor dipeptidyl-peptidase 4 (DPP4/CD26) of MERS-CoV have recently been demonstrated to reduce the interaction with the S glycoprotein [95]. In addition, different expression levels of ACE2 have been demonstrated to be positively correlated with SARS-CoV and NL63 (another human related respiratory coronavirus) infection [96]. Jia and colleagues have shown that a point mutation (L584A) in ACE2 can facilitate SARS-CoV entry into the host cell [97]. Hence, the genetic variation of *ACE2* between different populations may contribute to susceptibility to SARS-CoV-2. According to a recent report by Darbani and colleagues, 34 *ACE2* variants have been defined with importance for SARS-CoV-2 entry and infection [98]. The *ACE2* allele frequencies included six interaction-booster variants (S19P, I21V, K26R, T27A, N64K, and H378R) and eight interaction-inhibitor variants (E37K, N51D, K68E, F72V, M82I, G326E, Q388L, and P389H) which have been shown to vary significantly between populations. In addition, Darbani and colleagues showed that more than half of the variants were found in males, which may explain previous clinical observations showing higher mortality rates in males [99,100]. However, eight ACE2 variants located at the binding interface showed no disruption of the interaction between ACE2 and the RBD [101]. Cao and colleagues recently found two *ACE2* intron variants and ten other protein intron variants (located within or near the *ACE2* gene, three from *CLTRN*, five from *CA5B*, and two from an unknown gene) which showed association with higher ACE2 expression levels by genetic analysis of expression quantitative trait loci (eQTLs) [102]. Intriguingly, nine of twelve intron variants showed significantly higher allele frequencies in Asian populations when compared to others (African, European, and American). Notably, most of these intron variants were located at the *CLTRN* and the *CA5B* genes. Future study is required to clarify the correlation between the *ACE2* gene regulatory network and genetic variation.

Another systematic *ACE2* genetic analysis by Stawiski and colleagues identified nine *ACE2* variants which could increase susceptibility to SARS-CoV-2 and 17 *ACE2* variants which displayed protective roles on SARS-CoV-2 infection by structural computational analysis [103]. The missense variants of *ACE2* identified by recent studies, including key residues of ACE2 binding with SARS-CoV-2 RBD and residues with potential to affect binding, are summarized in Table 2. All of the *ACE2* missense variants in Table 2 are rare variants (<0.01 allele frequency), and most of the rare missense variants are distributed in European populations. However, based on our current knowledge, the *ACE2* rare missense variants in the population do not disrupt the interaction with the SARS-CoV-2 S glycoprotein. Hashizume and collegues identified seven *ACE2* missense variants which exist in Asian but not in American and European populations. They further demonstrated that these *ACE2* missense variants have a limited effect on SARS-CoV-2 infectivity in vitro [104]. In addition to the missense variants, overall ACE2 expression level is another factor which could affect SARS-CoV-2 transmissibility. According to current studies, the *ACE2* genetic variants with high allele frequencies are associated with the higher expression level of ACE2 in Asian populations. ACE2 expression is found to be significantly lower in North America, Europe, and Africa, in decreasing order [105]. Additionally, the difference in ACE2 expression is significantly correlated with the prevalence of the D614G variant across geographical regions [55]. Further biological study is required to confirm the relationship between the emergence of the D614G mutation with varying ACE2 expression levels.

**Table 2:** Summary of population frequencies of *ACE2* genetic variants related to SARS-CoV-2 S glycoprotein interaction.

	rs ID	Allele	Function Class	Global	African	Asian	European	American	Other	Potential Effect	Ref.
Key Residues of ACE2 Binding with RBD of SARS-CoV-2											
	rs73635825	A>G	S19P	0.00055	0.00326	0	0	0	0.00007	Interaction-booster	[28,98,103]
	rs781255386	T>C	T27A	0.00001	0	0	0	0.00009	0	Interaction-booster	[28,98,103]
	rs1348114695	C>T	E35K	0.00002	0	0.00006	0.00001	0	0	Interaction-inhibitor	[28,98,103]
	rs146676783	C>T	E37K	0.00005	0.00016	0	0.00006	0	0	Interaction-inhibitor	[28,98,103]
	rs766996587	C>T	M82I	0.00005	0.00025	0	0	0	0	Interaction-inhibitor	[28,98]
	rs143936283	T>C	E329G	0.00005	0	0	0.00008	0	0.00007	Interaction-inhibitor	[28,98]
	rs961360700	C>T	D355N	0.00001	0	0	0.00002	0	0	Interaction-inhibitor	[28,98,103]
Residues Potential Affect Binding											
	rs778030746	T>C	I21V	0.00002	0	0	0.00003	0	0	Interaction-booster	[98,103]
	rs1244687367	A>G	I21T	0.00001	0	0	0	0.00004	0	Interaction-booster	[98]
	rs756231991	C>T	E23K	0.00001	0	0	0.00002	0	0	Interaction-booster	[98,103]
	rs1299103394	T>C	K26E	0.00001	0	0	0.00001	0	0	Not characterized	-
	rs4646116	T>C	K26R	0.00323	0.00127	0.00079	0.00499	0.00229	0.0016	Interaction-booster	[98,103]
	rs1192192618	T>A	Y50F	0.00001	0	0	0.00001	0	0	Interaction-inhibitor	[98,103]
	rs760159085	T>C	N51D	0.00001	0	0.00008	0	0	0	Interaction-inhibitor	[98]
	rs1569243690	T>C	N51S	0.00001	0	0	0.00001	0	0	Interaction-inhibitor	[98,103]
	rs1325542104	T>C	M62V	0.00001	0	0	0.00001	0	0	Interaction-inhibitor	[98,103]
	rs1199100713	A>T	N64K	0.00005	0.00014	0	0.00007	0	0	Interaction-booster	[98,103]
	rs755691167	T>C	K68E	0.00002	0	0.00006	0	0	0	Interaction-inhibitor	[98,103]
	rs1256007252	A>C	F72V	0.00001	0	0.00003	0	0	0	Interaction-inhibitor	[98,103]
	rs763395248	G>A	T92I	0.00002	0	0	0.00003	0	0	Interaction-booster	[98,103]
	rs1395878099	T>G	Q102P	0.00003	0	0	0.00004	0	0.0001	Interaction-booster	[98,103]
	rs774621083	C>T	G220S	0.00002	0.00009	0	0.00002	0.00007	0	Interaction-inhibitor	[98]
	rs1448326240	A>C	H239Q	0.00001	0	0	0.00001	0	0	Interaction-inhibitor	[98]
	rs759579097	C>T	G326E	0.00001	0.00009	0	0	0	0	Interaction-inhibitor	[98,103]
	rs370610075	C>A	G352V	0.00001	0	0	0.00002	0	0	Interaction-inhibitor	[98,103]
	rs142984500	T>C	H378R	0.0001	0	0	0.00016	0	0	Interaction-booster	[98,103]
	rs751572714	T>A	Q388L	0.00002	0	0	0	0.00018	0	Interaction-inhibitor	[98,103]
	rs762890235	G>T	P389H	0.00003	0	0	0.00003	0.00014	0	Interaction-inhibitor	[98]
	rs1270795706	C>T	E467K	0.00002	0	0	0.00003	0.00004	0	Interaction-inhibitor	[98]

There are no single nucleotide polymorphism reports in other key RBD binding residues of ACE2 (Q24, F28, D30, K31, H34, D38, Y41, Q42, L45, L79, Y83, Q325, N330, K353, G354, R357, and R393. There are no single nucleotide polymorphism reports in other potential affect binding residues of ACE2 (A25, K31, N33, H34, D38, E75, Y83, H505, D509, R514, and Y515). The allele frequencies are obtained from 1000 Genome project, Exome Aggregation Consortium and Genome Aggregation Database on dbSNP on National Center for Biotechnology Information (NCBI) website (https://www.ncbi.nlm.nih.gov/snp/, accessed on 1 February 2021)

### 4.2. TMPRSS2 Genetic Variation and SARS-CoV-2 Infection

The *TMPRSS2* gene contains 14 exons located in chromosome 21. TMPRSS2 is mainly expressed on the luminal side of the prostate epithelium. The expression level is regulated by androgens, and overexpression of TMPRSS2 can be found in prostate cancer tissue [106]. In addition to its proteolytic activity, TMPRSS2 has been shown to be a critical helping factor in the fusion of influenza viruses and CoV into target cells [107,108]. A case-controlled genetic study identified two single nucleotide polymorphisms which are associated with high expression of TMPRSS2, and individuals who carry these polymorphisms were shown to be more susceptible to influenza virus infection [109]. TMPRSS2 is another essential protein for SARS-CoV-2 S glycoprotein priming [27]. The first question is whether the higher mortality rates in SARS-CoV-2 infected males are due to androgen-dependent TMPRSS2 expression. However, there is no difference in expression levels of TMPRSS2 between males and females in lung tissue [110]. The second question is whether genetic variation within *TMPRSS2* could affect its expression level, protein structure, and functions, further affecting individual susceptibility to SARS-CoV-2 infection. A systematic investigation of *TMPRSS2* variants identified 13 intron variants, two exon variants (coding regions), and six 3′UTR variants that can affect TMPRSS2 structure and function. rs12329760 and rs75603675 (both missense variants) potentially affect TMPRSS2 structure and post-translational modifications, respectively. Six 3′UTR variants (rs456142, rs462574, rs456298, rs12627374, rs12473206, and rs75036690) potentially affect the miRNA target activity [111]. Recently, another four variants, rs464397, rs469390, rs2070788, and rs38351, have been shown to be able to increase TMPRSS2 expression and show higher allele frequencies in European and American population when compared to Asian populations [112]. TMPRSS2 expression level is significantly lower in Africa due to genetic variability, which could possibly explain the lower number of reported infection cases in Africa [105].

Taken together, based on current evidence, genetic variations of *ACE2* and *TMPRSS2* are believed to affect individual susceptibility to SARS-CoV-2. However, a large scale clinical epigenetic study is needed to further confirm the effect of genetic variation on the susceptibility to SARS-CoV-2 infection.

### 4.3. DC/L-SIGN Genetic Variation and SARS-CoV-2 Infection

DC-SIGN is a C-type lectin receptor expressed on dendritic cells. A DC-SIGN related receptor called L-SIGN (or CD209L and DC-SIGNR) is expressed on lymph node and liver cells. The function of DC/L-SIGN is to recognize high mannose glycans on the cell and the pathogen surface [113,114]. Moreover, DC/L-SIGN binding with viral surface proteins can affect viral pathogenesis [115]. Notably, DC/L-SIGN can bind with the SARS-CoV S glycoprotein and facilitate virus transmission. Both L-SIGN and ACE2 are expressed on human type II alveolar cells which suggests that SARS-CoV can use both as entering receptors [116]. A previous study on SARS-CoV by Han and colleagues showed that seven glycosylation sites on the S glycoprotein play a vital role in DC/L-SIGN mediated virus entry [117]. Several studies have demonstrated that the allele frequency distribution of L-SIGN (CD209) promotor variant (rs4804803, -336A>G) is strongly associated with the pathogenesis of HIV-1, *Mycobacterium tuberculosis*, and Dengue infection [118,119,120]. Furthermore, Chan and colleagues showed that -336G is a protective allele for SARS-CoV infection [121]. Notably, the -336G allele distribution frequency is significantly lower in Asian populations than others (Asian (0.070) vs. African (0.426), South Asian (0.190), European (0.211), American (0.164), other (0.210), and global (0.244)). Therefore, it is speculated that the -336G allele may be positively associated with SARS-CoV-2 severity. Other than the -336G allele, the homozygosity of L-SIGN has also been found to play a protective role in SARS-CoV-1 infection [20]. Future case-controlled genetic studies are required to elucidate the correlation between *DC/L-SIGN* genetic variation and susceptibility to SARS-CoV-2 infection.

## 5. Conclusions and Perspectives

The pandemic of COVID-19 has caused more than 111 million confirmed cases and more than 2.4 million deaths globally as of 22 February 2021 since the first case was reported from Wuhan, China. The confirmed cases and deaths are rising quickly, and the fast evolution and transmission of SARS-CoV-2 has generated several particular mutations across geographic regions [122,123,124]. Since there were no vaccine and treatment-based selective pressures in the early pandemic, the host genetic variability could drive adaptive evolution by selecting for increased genetic diversity in SARS-CoV-2 across geographical regions. Mutations in the S glycoprotein have been shown to enhance viral transmissibility and immune escape ability, however, no current mutations increase viral pathogenicity or COVID-19 severity. The recent emergence of B1.1.7 (prevalent in the United Kingdom), B.1.351 (in South Africa), B.1.1.28 (in Brazil), and B.1.429 (prevalent in California, USA) show variants with several mutations in the S glycoprotein, especially within the RBD. Some mutations have been found to enhance viral infectivity (ΔH69/V70, N501Y, and P681H) or contribute to immune escape (ΔY144, ΔL242/244, E484K, L452R, and N501Y). Recent evidence has shown that K417N, E484K, and N501Y emerge in existing antibody selection pressure in vitro cell culture experiments, suggesting that those mutations are important for SARS-CoV-2 immune escape evolution [52]. Current COVID-19 vaccines seem to maintain intact neutralization activity for B.1.1.7, however, a remarkable decrease in neutralization activity for B.1.351 has been seen using sera from vaccinee and monoclonal antibodies [76]. The decrease of neutralizing activity is believed to be caused by the E484K mutation in S glycoprotein. SARS-CoV-2 carrying the aforementioned mutations, which have been found to co-occur with other variants and are circulating across geographic regions, should be monitored, as they contribute to decreased sensitivity to several clinically used monoclonal antibodies and human convalescent sera. There are different genetic nonsynonymous diversity patterns of SARS-CoV-2 across the world, possibly driven by genetic variation across human populations. To understand the role of host entry factors for SARS-CoV-2, future study should first focus on the correlation between the 21 genetic variants of *TMPRSS2* and susceptibility to SARS-CoV-2 in human populations. For DC/L-SIGN, future study is required to understand the correlation between genetic variants and the severity of COVID-19, especially focusing on -336G and the homozygous/heterozygous forms of L-SIGN. Continuing and enhancing surveillance, monitoring evolutionary changes of SARS-CoV-2 in different populations, and understanding the impact of mutations on viral transmissibility and immune escape ability are urgently needed to provide guidance on controlling and measuring transmission. Additionally, the acceleration of COVID-19 vaccine roll-out to the public is urgently needed to prevent SARS-CoV-2 evolution against the current vaccines.

## Data Availability

Sequences are available via the GISAID EpiCovTM database (https://www.gisaid.org/, accessed on 16 March 2021).
